# William Henry Spencer

**Published:** 1910-09

**Authors:** 


### Obituary Notices.

### WILLIAM HENRY SPENCER, M.A., M.D.Cantab.

*Fellow of the Linnæan and Royal Microscopical Society of London; Lecturer on Medicine, etc., at the Bristol University Medical School; Consulting Physician to the Bristol Royal Infirmary.*

Dr. Spencer was born at Oakhill, near Bath, on July 10th, 1837. He received his early education at Crawford House, Maidenhead, and later at Dr. Bigg's School, Devizes. His first choice of a profession was that of an engineer, and in 1854 he was apprenticed to Messrs. Craven's engineering works, Brighton. When he left in 1857 there was no part of an engine he had not made and put together, and he had himself driven an engine from Brighton to London.

Wishing to add to this technical knowledge an Arts degree, he then attended lectures at University College, London, with this end in view. Here he worked very closely, obtaining honour certificates in English Language and Literature and in Geology and Mineralogy. Shortly before the final examination his health broke down, and he had to give up his studies and put himself under the care of Dr. Edwin Martin at Weymouth to recoup.

Dr. Martin was a man far in advance of his time in the treatment of nervous diseases, and it was the close intercourse with him, and the realization that their minds worked so much on the same lines, that decided Dr. Spencer to give up engineering and prepare for the medical profession, a decision he never regretted.

Having regained his health, in 1858 he entered Jesus College, Cambridge, to prepare for his medical degree, attending Addenbrook's Hospital for the practical work.

In July, 1859, though still an undergraduate, he married, and in consequence migrated to Downing College, where special arrangements were made for married undergraduate fellow-commoners.

In 1862 he took his B.A. in the Natural Science Tripos of that year.

Some of the arrangements for the practical part of the Natural Science Tripos were at that period primitive in the extreme mutton chops not infrequently being provided for the candidates to dissect for demonstrating the tissues of the human body. This gave rise to a scathingly satirical criticism of the practical part of these examinations which Dr. Spencer contributed as an article to *The British Medical Journal*, an article which he was told, many years after, sounded the knell of these primitive proceedings. After taking his degree, and while continuing his medical training, Dr. Spencer began coaching for Natural Sciences in Cambridge. For this purpose he rented two stables in Trumpington Street, converting them into the first Physiological Laboratory in the University. Unfortunately Dr. Spencer had to give up his connection with this in 1865, as it was necessary for him to go to London to complete his preparation for his M.B., and he entered St. Mary's Hospital, Paddington, as Clinical Clerk to his friend the late Dr. Gibson. Here he still continued coaching in Medicine and Natural Sciences, in combination with his two friends Hanbury and Valentine, who took Mathematics and Classics respectively, also lecturing on Physiology at the Charing Cross Medical School. In 1866, he took his M.A.. Family affairs took him up to Newcastle-on-Tyne, in 1868, where he took the post of medical tutor to the Royal Infirmary Medical School, now the Durham University Medical School.

In 1872 he took his final M.B. at Cambridge. The same year he went to Clifton, where there appeared to be an opening for a physician, it having been his wish to settle within reach of his old home and his relations at Oakhill, Somerset.

Shortly after this there was a vacancy on the staff of the Bristol Royal Infirmary, when he was appointed Assistant-Physician, and in time, as vacancies occurred, full Physician, Dean of the Faculty, and, before leaving Clifton, Consulting Physician to this Institution. He loved his work there, and to the end of his days the Bristol Royal Infirmary, and those with whom he worked, were very near his heart.

Soon after his appointment to the Infirmary he became Lecturer on Physiology, later on Pathology, and the Principles and Practice of Medicine at the Bristol Medical School. This school had been in existence for many years, and Dr. Spencer, with his experience of teaching and love of organisation took the work up keenly. Soon afterwards the Bristol University College was started, Dr. Spencer being one of the principal promoters, and whilst he remained in Clifton he was on the Executive Committee with Professor Jowett, Sir William Ramsay, Professor Alfred Marshall, and other prominent men. All who worked with him know with what keen interest and indefatigable zeal Dr. Spencer laboured for the welfare of the Bristol Royal Infirmary, the Medical School, and University College. At some period during his connection with these Institutions he represented Bristol on the Council of the British Medical Association.

In 1878 Dr. Spencer took his M.D., his thesis for this being "On the Tendency of Disease to cause Disease," and in 1888 he became a Member of the College of Physicians, London.

In 1874 Dr. Spencer brought out and gave to his profession an instrument he had long been perfecting, a new form of binaural and differential stethoscope. This stethoscope was the parent of all the double stethoscopes now in use, and was described when it came out as "a perfect scientific instrument." One of its chief features was the hollow joint, whereby the sounds were conveyed to both ears at the same time, and by compressing the flexible rubber tubing below the joint first one side of the chest and then the other could be listened to and compared without removing the cups from the ears. An instrument very similar, but more clumsy in form and without the hollow joint, was shortly after this brought out in Germany. It is now almost the exception to find the old form of stethoscope used, yet there are very few who associate Dr. Spencer's name with the binaural one.

It is as long ago as 1872 that Dr. Spencer obtained an appointment on the staff of the Bristol Royal Infirmary, when he succeeded the late Dr. Ebenezer Ludlow as Assistant-Physician in charge of the medical out-patients. In the following year he became Physician, and for thirteen years carried out the duties of Honorary Physician with indefatigable zeal. He was a good physician and a good colleague, so much so that he was appointed by the staff to act as Dean of the Faculty.

In 1874 he became one of the original founders of the Bristol Medico-Chirurgical Society. The first meeting of this Society was convened by a circular, dated February 20th, 1874, signed by Nelson C. Dobson, R. Shingleton Smith and W. H. Spencer, when those present constituted the nucleus of the future Society, and Dr. F. Brittan, the first President, gave an address on "Germ Theories." Dr. Spencer took a warm interest in the Society during its early years; he contributed many papers, joined in various discussions, and became president of the Society in the year 1885, when he gave a very learned, historic, but somewhat theoretical discourse on "Pyrexia, a Retrospect, a Review, and a Forecast."

In 1888 Dr. Spencer took advantage of what seemed an exceptional opportunity for stepping into a consulting practice at St. Leonards-on-Sea, taking the place of the late Dr. Arthur Gamgee there. Here also were exceptional conditions for carrying out his treatment for consumption by inhalation, on which for some years he had been wishing to specialise, and on which he had written much.

Here he perfected and finally patented the "Arema Vaporiser" and the special inhalants to be used in it, publishing a treatise on *Consumption: its Nature and Treatment*, explaining the use of the vaporiser and the application of the inhalants. Both at Bristol and St. Leonards-on-Sea he had some good results from this treatment, the accounts of which he published in the medical journals. This vaporiser was adopted at the Brompton Hospital for Consumption and at many sanatoria in different parts of the country. Vaporisers in many forms have been brought out since, but none carry out so completely as does this, with its water bath regulating the temperature of the vaporants, the scientific and chemical conditions necessary for vaporising those volatile oils to such a degree that they, when inspired, can readily pass into every part of the interior of the lungs.

As most people must realise, inventors rarely, if ever, benefit by or even get the credit for their inventions. A fertile and inventive brain seldom goes with the possession of the means necessary for making a commercial success of an invention. It is often those who can supply the money whose name is associated with, and who gain financially by, the bringing out of anything new. Dr. Spencer was no exception to this rule. The binaural stethoscope he gave to the profession, the vaporiser he patented. Of this latter the small financial gain that accrued to him went but a small way toward meeting the expenses of perfecting and patenting the instrument.

The expenses incurred by this, with other drains on his resources, and the fact that the practice he hoped to enjoy had followed its maker, finally broke up his home.

After this he did little practice, but spent a good deal of his time visiting the south coast health resorts and the many sanatoriums for the treatment of consumption, and introducing his inhalation treatment, hoping in time to start a sanatorium of his own.

In July, 1909, he felt the first serious symptoms of the disease to which he finally succumbed—sclerosis of the spinal cord, involving general paralysis. From this time he became a partial invalid, and died in Oxford on May 27th, 1910, at the age of 72.

PUBLICATIONS.

*Elements of Qualitative Chemical Analysis.*—London: Macmillan. 1866.

*The Tendency of Disease to cause Disease, illustrated by the Pathogeny of Diseases of the Heart and Lungs.* M.D. Thesis.—London: H. K. Lewis. 1872.

"The Chemical and Therapeutic Relations of Salicine and Salicylic Acid."—*Tr. Bristol M.-Chir. Soc.*, 1874, i, 16.

"On a New Form of Stethoscope in its Relations to the Theory and Practice of Auscultation."—*Brit. M. J.*, 1874, i, 409.

"Opium, Aconite and Salicylic Acid in Acute Rheumatism."—*Ibid.*, 1878, ii, 163.

"Case of Idiopathic Inflammation of the Spinal Dura Mater."—*Lancet*, 1879, i, 836.

"On Giving Rest to the Digestive Organs by the use of Peptonised Food." —*Bristol M.-Chir. J.*, 1884, ii, 32.

"Two Cases of Gastric Ulcer treated by Peptonised Enemata."—*Ibid.*, p. 41.

"A New Neurosis (Thomson's disease)."—*Ibid.*, p. 159.

"Pyrexia: a Retrospect, a Review, and a Forecast" (President's Address). —*Ibid.*, 1885, iii, 217.

"The Scope of Medicine as a Department of Knowledge: Health and Disease."—*Med. Times and Gaz.*, 1885, ii, 627.

"Lectures on the Principles and Practice of Medicine."—*Ibid.*, pp. 627, 663.

"Cases Illustrating the Antiseptic and Antipyretic Treatment of Phthisis." —*Ibid.*, 1888, i, 184.

*A New Method of Inhalation for the Treatment of Diseases of the Lungs.* 1895.

*Consumption: its Nature and Treatment.* 1900.

